# Occurrence and Antimicrobial Resistance of *Arcobacter* spp. Recovered from Aquatic Environments

**DOI:** 10.3390/antibiotics10030288

**Published:** 2021-03-10

**Authors:** Sonia Sciortino, Pietro Arculeo, Vincenzina Alio, Cinzia Cardamone, Luisa Nicastro, Marco Arculeo, Rosa Alduina, Antonella Costa

**Affiliations:** 1 Food Microbiology Section, Istituto Zooprofilattico Sperimentale della Sicilia A. Mirri, Via G. Marinuzzi 3, 90129 Palermo, Italy; pietro.arculeo@izssicilia.it (P.A.); vincenzina.alio@izssicilia.it (V.A.); cinzia.cardamone@izssicilia.it (C.C.); luisa.nicastro@izssicilia.it (L.N.); antonella.costa@izssicilia.it (A.C.); 2 Department of Biological, Chemical and Pharmaceutical Sciences and Technologies, University of Palermo, Viale delle Scienze, Bd. 16, 90128 Palermo, Italy; marco.arculeo@unipa.it

**Keywords:** *Arcobacter butzleri*, water samples, multiplex PCR, antibiotic susceptibility, *tetO*, *tetW*

## Abstract

*Arcobacter* spp. are emerging waterborne and foodborne zoonotic pathogens responsible for gastroenteritis in humans. In this work, we evaluated the occurrence and the antimicrobial resistance profile of *Arcobacter* isolates recovered from different aquatic sources. Besides, we searched for *Arcobacter* spp. in seaweeds and the corresponding seawater samples. Bacteriological and molecular methods applied to 100 samples led to the isolation of 28 *Arcobacter* isolates from 27 samples. The highest prevalence was detected in rivers followed by artificial ponds, streams, well waters, and spring waters. Seaweeds contained a higher percentage of *Arcobacter* than the corresponding seawater samples. The isolates were identified as *Arcobacter butzleri* (96.4%) and *Arcobacter cryaerophilus* (3.6%). All the isolates showed a multi-drug resistance profile, being resistant to at least three different classes of antibiotics. Molecular analysis of genetic determinants responsible for tetracycline resistance in nine randomly chosen isolates revealed the presence of *tetO* and/or *tetW.* This work confirms the occurrence and the continuous emergence of antibiotic-resistant *Arcobacter* strains in environmental samples; also, the presence of quinolone-resistant *Arcobacter* spp. in aquatic sources used for water supply and irrigation represents a potential risk for human health.

## 1. Introduction

The members of the *Arcobacter* genus are Gram-negative, slender, spiral-shaped rods and belong to the family Campylobacteraceae [1]: they are distinguished from *Campylobacter* genus by their ability to grow in aerobic conditions and at lower temperatures from 15 to 30 °C [2].

*Arcobacter* spp. are emerging entero-pathogens that can be isolated worldwide from different aquatic matrices, such as lakes and rivers [3,4,5,6], groundwater [7,8], wastewater [9,10,11], drinking water [12], seawater [13,14], and food of both animal and non-animal origin [15,16,17,18]. Thus, food and water are considered the main vehicle of the pathogen [19,20]. *A. butzleri*, *A. cryaerophilus*, and *A. skirrowii* have been associated with animal and human infections and *A. butzleri* has been classified as a serious hazard to human health by the International Commission on Microbiological Specifications for Foods in 2002 [21] and is often correlated with bacteremia, gastroenteritis, and watery diarrhea in humans [22,23,24]. Alarming outbreak episodes of *A. butzleri* have been reported in a nursery and primary school in Italy [25], associated with groundwater, which served as the drinking water source in Idaho, USA [7], and related with the consumption of water contaminated with wastewater from sewage treatment plants [8]. The presence and the persistence of *A. butzleri* in the environment could be dependent upon its ability to form biofilms that allow its survival in various conditions, favoring bacterial diffusion and transmission within the different food chains [26].

*A. butzleri* is known to contain numerous virulence genes and to cause intestinal and extra-intestinal infections, that are often self-limited [27,28]. *A. butzleri* infection can be treated with antibiotics, i.e., β-lactams, fluoroquinolones, macrolides [28]. However, *Arcobacter* species frequently display a multidrug-resistant profile, hampering the antibiotic treatment of *A. butzleri* infections. Recent studies have indicated an increase of resistance against fluoroquinolones as well as tetracycline of *A. butzleri* and *A. cryaerophilus* isolates from food and aquatic sources [27,29,30].

The foods that usually are consumed raw, such as vegetables, have been found to carry *A. butzleri* and strains isolated from these matrices have been demonstrated to possess many virulence and antibiotic resistance genes [26]. The high prevalence of antimicrobial resistance among bacteria may be dependent upon the use of antibiotics in animal production and human medicine [31,32]. Aquatic environments and sea animals are considered as reservoirs of antibiotic resistance genes [33,34,35] and *Arcobacter* spp. can be isolated worldwide from different aquatic matrices, such as lakes and rivers [3,4,5,6]. In recent years, an increasing number of scientific papers concerning *Arcobacter* focused on the growing importance of this emerging entero-pathogen [36].

Recently, the antibiotic-resistance profile and the genomic diversity of this pathogen were exploited by comparing 49 *A. butzleri* strains isolated from various environments and samples [37]. All isolates were resistant to nalidixic acid, followed by cefotaxime, ampicillin, levofloxacin, ciprofloxacin, and erythromycin. Comparison of the antibiotic-resistance profile and the genome sequences revealed that *A. butzleri* contains many genes coding for efflux pumps and other antibiotic resistant determinants, for example, quinolone resistance is due to the mutation Thr-85-Ile of the *gyrA* gene [29].

This study aimed to detect and identify *Arcobacter* spp. from different environmental water sources using bacteriological and molecular methods, to determine the antibiotic resistance profile of the isolates and to investigate the antibiotic genetic determinants providing tetracycline resistance.

## 2. Results

### 2.1. Isolation and Identification of Arcobacter Species

Twenty-eight *Arcobacter* strains were isolated out of 100 samples (28%). Specifically, 9 *Arcobacter* strains were found in 11 rivers (81.8%), 6 in 8 artificial ponds (75%), 2 in 5 streams (40%), 2 in 20 well waters (10%), and 1 in 17 spring waters (5.8%) used for water supply (Table 1). All the analyzed drinking water samples were negative. However, *Arcobacter* spp. was identified in a sample of non-chlorinated source water (spring water) used for drinking.

Furthermore, *Arcobacter* spp. was searched in seaweeds belonging to the genus *Enteromorpha* and in the corresponding seawater samples, collected from six independent locations of the northern coast of Sicily. Interestingly, while 3 *Arcobacter* spp. were isolated from seawater samples (50%), 5 out of 6 algal samples were positives to *A. butzleri* (83.3%) (Table 2).

**Figure 1:**
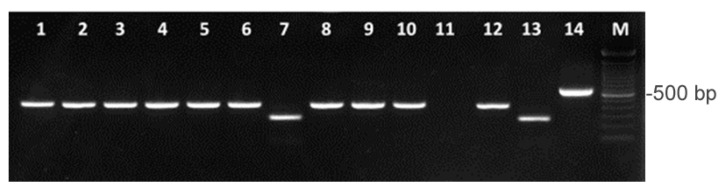
Multiplex PCR results of ten *Arcobacter* isolates. Lanes 1–6: *A. butzleri*; lane 7: *A. cryaerophilus*; lanes 8–10: *A. butzleri*; lane 11: negative control; lane 12: positive control (*A. butzleri*, NCTC 12481); lane 13: positive control (*A. cryaerophilus*, NCTC 11885); lane 14: positive control (*A. skirrowii*, NCTC 12713); lane M: 100 bp DNA Ladder (Invitrogen).

**Figure 2:**
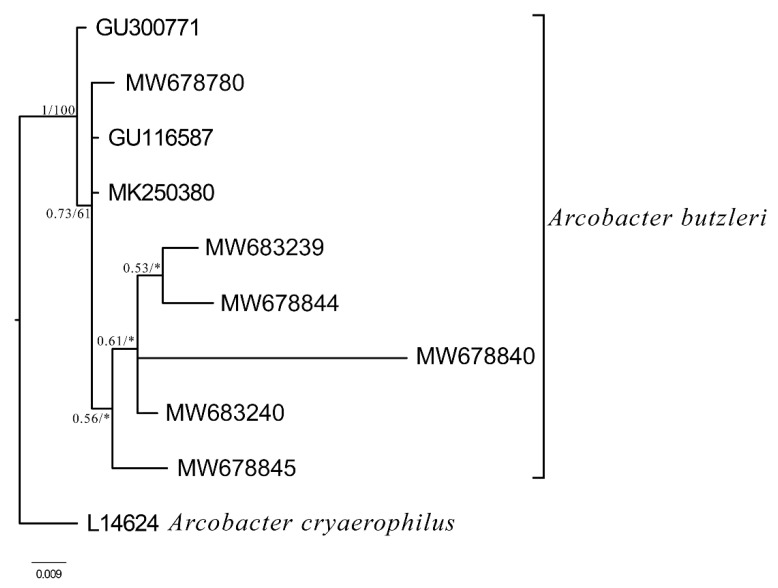
Bayesian phylogram of *A. butzleri*. based on the 358 bp fragment of the 16S rDNA. Sample of *A. cryaerophilus* (A.N. L14624) was used as outgroup to root the tree. Node statistical support is reported as nodal posterior probabilities (Bayesian Inference of phylogeny, BI)/bootstrap values (Maximum Likelihood, ML). Asterisks indicate a bootstrap support value lower than 50. GenBank Accession Numbers reported in bold refer to novel sequences obtained in this study.

Multiplex PCR (mPCR) demonstrated that 27 out of the 28 isolates corresponded to *A. butzleri* (96.4%) and only one to the *A. cryaerophilus* (3.8%) (Figure 1). *A. butzleri* and *A. cryaerophilus* were co-isolated from a water sample of an artificial pond populated by aquatic birds. *A. skirrowii* was not detected in any of the samples. PCR amplicons were sequenced and BLAST alignment revealed a 98–99% identity with *A. butzleri* 16S rDNA gene and 99% identity with *A. cryaerophilus* 23S rDNA gene. Phylogenetic tree of the sequences of the amplification product obtained by *A. butzleri* (Figure 2) indicated a well-structured clade.

### 2.2. Antimicrobial Susceptibility Testing

**Figure 3:**
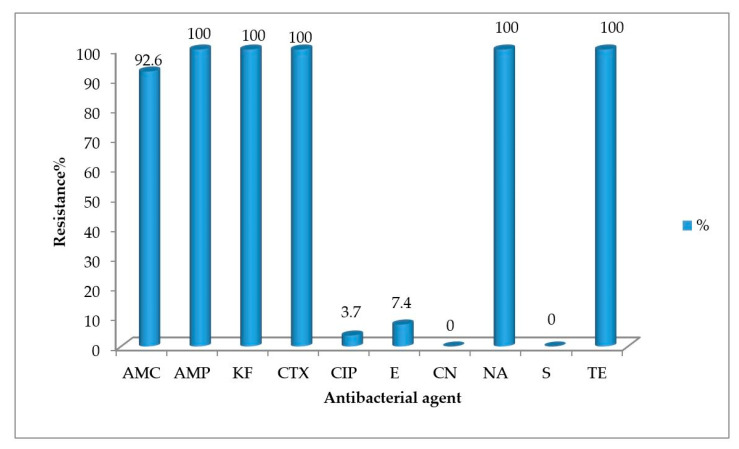
The percentage of antibiotic resistance of *A. butzleri* isolates (AMC: amoxicillin-clavulanic acid, AMP: ampicillin, KF: cefalotin, CTX: cefotaxime, CIP: ciprofloxacin, E: erythromycin, CN: gentamycin, NA: nalidixic acid, S: streptomycin, TE: tetracycline). The resistance percentage was calculated as the ratio of number of antibiotic resistant isolates divided by the total number of *A. butzleri* isolates (*n* = 27).

**Figure 4:**
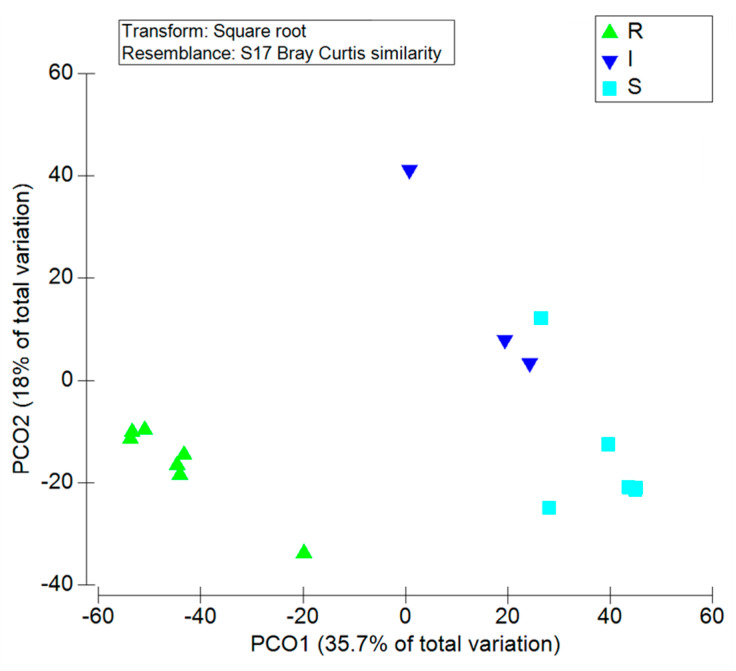
Principal coordinates analysis of the resistant, intermediate, and sensitive isolates of *A. butzleri.* R: resistant; I: intermediate; S: susceptible.

All *A. butzleri* isolates were susceptible to gentamicin (CN) and streptomycin (S), and resistant to ampicillin (AMP), cefalotin (KF), cefotaxime (CTX), nalidixic acid (NA), and tetracycline (TE). *A. butzleri* strains were resistant to amoxicillin-clavulanic acid (AMC), erythromycin (E), and ciprofloxacin (CIP) at the rate of 92.6%, 7.4%, and 3.7%, respectively (Figure 3 and Table 3). *A. cryaerophilus* strain showed resistance to 9 out of the 10 tested antibiotics and sensitivity to gentamicin (CN). Multidrug-resistance, defined as resistance to three or more tested antibiotics, was observed in all the isolates. PCoA (Figure 4) indicated 3 distinct clusters, containing the resistant, the intermediate, and the sensitive isolates.

### 2.3. Analysis of the Quinolone and Tetracycline Resistance Genes

The search for tetracycline resistance genes by PCR in the genome of nine randomly chosen tetracycline-resistant isolates revealed that all carried *tetW* or *tetO*, even simultaneously (Table 4). No isolates contained the *tetA* gene. The two ciprofloxacin-resistant isolates were tested for the presence of the resistance gene *qnrS* by PCR and sequencing of the *gyrA* amplification product. The sequence of the *gyrA* gene did not show the mutation associated with a quinolone resistance phenotype, nor the PCR of *qnrS* gave the amplicon of the expected size (data not shown).

## 3. Discussion

This study enlarges the knowledge on the spread and the antibiotic resistance profile of the emerging enteropathogen *Arcobacter* in water samples. Even if the *Arcobacter* genus is widespread in various environments, water may play an important role in its transmission to animals and humans. Our results showed a higher prevalence of *Arcobacter* in the surface waters (streams, rivers, ponds) and a very low occurrence in waters dedicated to human consumption (well water, spring water, and drinking water) in accordance with other reports which correlated the presence of *Arcobacter* species with fecal contamination [9,38]. Specifically, in this study, *A. butzleri* was the predominant species and was isolated in 81.8% of the river water samples and 75% of the artificial ponds where aquatic animals (birds, turtles) lived. *A. cryaerophilus* was co-isolated together with *A. butzleri* from an artificial pond, while *A. skirrowii* was never detected in any of the samples. Indeed, *A. butzleri* is the most frequent species isolated from different water samples such as 23% in river water [5] and 55.1% in freshwater, seawater, and sewage samples [9]. *A. butzleri* was detected in the creek (26.31%) and stream water samples (18.36%) and not isolated from ponds and drinking water samples in the Kars region [39]. *A. butzleri* was also isolated from surface water samples (25.6%) and treated wastewater samples (77.9%) in southwestern Alberta, Canada [4]. Talay et al. (2016) reported a prevalence of 35.7% from various aquatic sources including sewages, rivers, and spring waters, of which 34% were positive for *A. butzleri* [11]. In Sicily, *Arcobacter* spp. was detected from surface waters and in estuarine waters of rivers [13,40]. Water birds (ducks, geese, etc.) can be reservoirs of *Arcobacter* [41,42] and this would probably explain the higher incidence of isolation in the artificial ponds inhabited by these animals. The waters collected, in this study, from the ponds contained *Arcobacter*, while the spring water samples, did not. Besides, the samples of drinking waters here analyzed were negative; however, *A. butzleri* was isolated from a sample of non-chlorinated source water (spring water) used for human consumption, considered as drinkable water for the absence of the fecal contamination markers, i.e., *Escherichia coli* and enterococci. The presence of *Arcobacter* was reported in drinking water in Turkey [43] and in treated water samples in Malaysia [44] with percentages of 3% and 11.1%, respectively. Cases of *Arcobacter* outbreaks associated with contaminated water have been documented worldwide [7,12,26]. Because *Arcobacter* is sensitive to chlorination [7,45], its isolation in drinking water might indicate either ineffective chlorination or recontamination after chlorination [43]. Due to its ability to form biofilms [2,46], chlorination could not suppress *Arcobacter* colonization, in fact, biofilms of this strain were found in drinking water distribution pipes [47].

Seawater is also mentioned as a potential source of *Arcobacter* spp. In our study, *A. butzleri* was identified in three out of 21 samples of seawater examined (14.2%). Interestingly, we found that algae of the genus *Enteromorpha,* collected together with seawater samples, were more frequently colonized by *A. butzleri* with a value of 83.3% than the corresponding seawater samples (50%) (Table 2). Algae could represent a suitable microhabitat for *Arcobacter* and other bacteria. Previous studies reported that *A. butzleri* was more abundant in seawater and plankton samples collected from the Straits of Messina, Italy, when associated with plankton than free-living [13,36].

Although the illness caused by Arcobacter can be self-limited, antibiotics, such as aminoglycosides, tetracyclines, and fluoroquinolones are recommended as the drugs of choice for the treatment for *Arcobacter* infection in human and animals [19,48]. However, strains resistant to these antibiotics have been detected in food and water sources [18,45,46,47]. The resistance of *A. butzleri* isolates to β-lactams is widespread in water sources as well as in other environments [49]. The resistance of *Arcobacter* spp. to cephalosporins is known; in fact, these antibiotics are commonly used for the isolation of Campylobacteraceae in selective media [50]. Recently, the genetic determinants associated with the resistance mechanisms have been exploited by comparing the genome sequences of 49 strains [37].

In our results, multidrug resistance was observed in all *Arcobacter* isolates tested. Precisely, all *Arcobacter* isolates were resistant to tetracycline and nalidixic acid, and the β-lactam antibiotics, ampicillin, cefalotin, cefotaxime, and AMC (except two isolates). Only three *A. butzleri* isolates, collected from seawater, seaweed, and a river were erythromycin- and ciprofloxacin-resistant and one *A. butzleri* isolate was ciprofloxacin-resistant; the *A. cryaerophilus* isolate displayed resistance to all the tested antibiotics, except gentamycin. Šilha et al. [49] reported that *A. cryaerophilus* strains collected from water sources were sensitive only to tetracycline and gentamicin.

It is interesting to note that the majority of studies report that *A. butzleri* isolates are highly susceptible to tetracycline, so that it can be used for human *Arcobacter* infections [50,51], while out study demonstrated an increasing resistance of the isolates.

Tetracycline resistance is dependent upon more than 40 genes (*tet* genes). Our molecular analysis demonstrated that among three different genetic determinants known to be involved in tetracycline resistance (*tetA*, *tetO,* and *tetW*) results, all the tetracycline-resistant isolates carried *tetO* and *tetW,* even together, while no isolates contained *tetA*.

The *tetO* and *tetW* genes are found more frequently than other *tet*-genes (e.g., *tetA*) in commensal bacteria isolated from fecal and water samples [52]. The fact that four out of nine isolates contained two genetic determinants is worrying. *tetO* and *tetW* genes confer ribosomal protection from the inhibiting effect of tetracycline [53,54,55], and they appear to be promiscuous in environmental organisms through different transfer mechanisms [56,57]. All isolates were resistant to nalidixic acid and two to ciprofloxacin. The search for *qnrS* and the sequencing of the *gyrA* gene did not explain the resistance to ciprofloxacin. To the best of our knowledge, the quinolone-resistance of *A. butzleri* is due to the mutation Thr-85-Ile of the *gyrA* gene [37]. Thus, further investigation is necessary to understand the molecular basis of quinolone resistance in these isolates.

The results obtained in this work show that aquatic sources can be a vehicle of potential pathogenic *Arcobacter* spp. Water is a likely key component to *Arcobacter* transmission, particularly, in intensive farming operations where water is consumed by the animals or in processing plant [58] or used for vegetable products [59] and water contamination could be due to feces of livestock animals [60] and farm effluents [9,61]. These bacteria can adapt and survive promptly in environmental waters, such as rivers, canals, and irrigation water; indeed, *Arcobacter* may survive in environmental waters, replicate at refrigeration temperatures [2], and develop the viable nonculturable state, thus representing a potential risk for human health [62,63].

To better assess the risks for human health, it is important to deepen these investigations and to search *Arcobacter* strains in non-chlorinated water that can be used for water supply and for irrigation of raw consumed vegetables.

## 4. Materials and Methods

### 4.1. Sampling

One hundred samples (Table 1) including rivers (*n* = 11), streams (*n* = 5), artificial ponds (*n* = 8), well waters (*n* = 20), spring waters (*n* = 17), drinking waters (*n* = 12), and seawater (*n* = 21), were collected between February and December 2017 in all around Sicily; in addition, seaweed samples (*n* = 6) were taken in consideration.

All the water samples, collected in 1 L sterile flasks, were transported under cold storage temperature (4 °C) to the laboratory and analyzed within 24 h.

### 4.2. Isolation of Arcobacter

For the isolation of *Arcobacter* spp. from water samples, the protocol reported in Collado et al. 2008 [9] was followed. Specifically, 200 mL of water were filtered using a 0.45 µm nitrocellulose membrane filter (Sartorius). For *Arcobacter* isolation from seaweeds, 10 g were weighed. Filters or seaweeds were placed into sterile bags containing 30 or 90 mL, respectively, of *Arcobacter* enrichment broth (Oxoid, UK) added with cefoperazone, amphotericin B, and teicoplanin (CAT) selective supplement (SR0174, Oxoid, UK) and incubated a 30 °C for 48 h under aerobic condition. After incubation, 200 µL of the broth were then dropped onto the surface of 0.45 µm nitrocellulose membrane filter (Sartorius), placed onto two selective agar plates: trypticase soy agar (TSA) plus 5% Laked Horse blood (Oxoid) with CAT and modified charcoal cefoperazone deoxycholate agar (mCCDA) supplemented with CAT. Plates were incubated at room temperature for 30 min and after removal of the filters, incubated at 30 °C for 48 h to 72 h under aerobic conditions [16]. Subsequently, suspected colonies grown within the filter area with a diameter between 0.5 and 2 mm, were picked, subcultured onto blood agar, and incubated at 30° C for 48 h [6]. Presumptive identification tests (Gram staining, catalase, oxidase, urease tests, and motility) were performed on at least five suspected colonies. The isolates referable as *Arcobacter* genus (Gram negative, spiral- shaped, motile, oxidase and catalase positive, urease negative), were stored in 20% (*v*/*v*) nutrient broth–glycerol at −80 °C, for subsequent molecular identification.

### 4.3. Identification of Arcobacter Species by Multiplex PCR

DNA was extracted by using the protocol previously reported [15] and utilized as template in a multiplex PCR assay to amplify the 16S and 23S rRNA genes in order to obtain a specific and simultaneous identification of *A. butzleri*, *A. cryaerophilus*, and *A. skirrowii* [64]. The selected primers amplified a 401-bp fragment from *A. butzleri*, a 257-bp fragment from *A. cryaerophilus*, and a 641-bp fragment from *A. skirrowii*. The amplification products were then separated by electrophoresis on 1.5% agarose gels, stained by SYBR Safe DNA gel stain, at 100 V for 40 min, and the bands were visualized under UV transilluminator (GelDoc-It, UVP Cambridge, UK). DNA from reference strains *A. butzleri* (NCTC 12481), *A. cryaerophilus* (NCTC 11885) and *A. skirrowii* (NCTC 12713) were used as positive controls and sterile distilled water was used as negative control.

### 4.4. Identification of Arcobacter Species by Sequence Analysis

The PCR products of *Arcobacter* spp. were purified using Illustra GFX PCR DNA and Gel Band Purification kit (GE Healthcare) following the manufacturer’s instructions for sequencing analysis. The purified products were sent to Macrogen Company (Amsterdam, Holland) for Sanger sequencing. The identification was performed by the alignment of the sequences against a reference database (GenBank). Novel sequences are available online in the GenBank™ database under the accession numbers MW678780, MW683239, MW678840, MW678844, MW678845, MW683240. The software packages MrBayes v. 3.2.7) [65] and MEGAX [66] were used for inferring phylogenetic relationships through Bayesian inference of phylogeny (BI) and maximum likelihood analysis (ML). As support measures for the nodes, bootstrap values were calculated with 1000 replicates in the ML trees, whereas in the BI tree, the posterior probability values were reported. PartitionFinder v. 1.0.1 [67] was used to choose the best evolutionary model following the Akaike information criterion (AIC). For the 16S rDNA fragment, the general time-reversible model of evolution with gamma-distributed rate variation among sites (GTR+Γ; nst = 6) was used for both the BI and ML analyses. Six sequences were used as representatives.

### 4.5. Antimicrobial Susceptibility Testing

Antimicrobial susceptibility testing of the *Arcobacter* isolates against 10 antibiotics was performed on Mueller-Hinton agar (Oxoid) by disk diffusion method according to the guidelines of the Clinical and Laboratory Standard Institute (CLSI) for *Campylobacter jejuni/coli*. Amoxicillin-clavulanic acid (AMC, 30 µg), ampicillin (AMP, 10 µg), cefalotin (KF 30 µg), cefotaxime (CTX, 30 µg), ciprofloxacin (CIP, 5 µg), erythromycin (E, 10 µg), gentamicin (CN, 10 μg), nalidixic acid (NA 30 µg), streptomycin (S, 10 µg), tetracycline (TE, 30 μg) disks (OXOID, UK) were used. Fluoroquinolone ciprofloxacin, tetracyclines, and aminoglycosides, such as gentamicin, kanamycin, and streptomycin, represent the most common antimicrobial agents for treatment of *Arcobacter* infections, because of high frequency of susceptibility towards them. The isolates were sub-cultured on blood agar base (Oxoid) and after incubation at 37 °C in aerobic condition for 24 h, a 0.5 MacFarland bacterial suspension was prepared in saline solution and spread on Mueller-Hinton agar. The plates were incubated at 37 °C for 24 h under aerobic condition. Then, the diameters of inhibition zones were measured and the isolates were classified as resistant (R), susceptible (S), or intermediate (I). The experiments were performed in duplicate. The *A. butzleri* LMG 10828T (RM4018) strain was used for comparison purposes. Principal coordinate analysis (PCoA) was performed using the software package PRIMER 6 [68]. The analyses were based on Bray–Curtis distance matrix.

### 4.6. Analysis of the Quinolone and Tetracycline Resistance Genes

DNA was extracted from 9 samples and utilized as template to amplify the genes coding for products responsible for the resistance to tetracycline (*tetA*, *tetO*, and *tetW*) and to quinolones (*qnrS* and *gyrA*) using the primer pairs reported in Table 5. Amplicons were detected using a 6% polyacrylamide non denaturing gel in TBE 0.5X, except *gyrA* amplicon that was detected using a 1.5% agarose gel. The presence of the expected amplification product was considered as a positive sample. All the PCR products were sequenced.

## 5. Conclusions

In the present study, we evaluated the presence of *Arcobacter* species in different environmental water sources. Our results showed the spread of this important zoonotic agent in the environment, which can be considered a potential risk for food safety. The determination of *Arcobacter* in water sources might be important to better understand the epidemiology and the ecology of these bacteria.

All the *Arcobacter* isolates displayed an alarming multi-drug resistance. Molecular analysis of genetic determinants responsible for tetracycline resistance in nine randomly chosen isolates revealed the presence of *tetO* and/or *tetW.* This work confirms the occurrence and the continuous emergence of antibiotic-resistant *Arcobacter* strains in environmental samples; besides, the presence of antibiotic-resistant *Arcobacter* spp. in aquatic sources used for water supply and irrigation represents a potential risk for human health.

## Figures and Tables

**Table 1:** Prevalence and molecular identification of *Arcobacter* spp. in the examined samples.

Sample (Source)	N. of Samples	*Arcobacter* spp. (%)	*A. butzleri*	*A. cryaerophilus*
Rivers	11	9 (81.8)	9	
Ponds	8	6 (75)	5	1
Streams	5	2 (40)	2	
Well water	20	2 (10)	2	
Spring water	17	1 (5.8)	1	
Drinking water	12	0	-	
Seawater	21	3 (14.2)	3	
Seaweeds	6	5 (83.3)	5	
Total	100	28 (28%)	27 (96.4%)	1 (3.6%)

**Table 2:** *Arcobacter* spp. in the examined seawater and seaweeds.

Sample Number	Area of Origin	Type of Sample	*Arcobacter* spp.
1	Messina	Seawater	ND
		Seaweed	*A. butzleri*
2	Palermo	Seawater	ND
		Seaweed	ND
3	Palermo	Seawater	*A. butzleri*
		Seaweed	*A. butzleri*
4	Messina	Seawater	ND
		Seaweed	*A. butzleri*
5	Palermo	Seawater	*A. butzleri*
		Seaweed	*A. butzleri*
6	Messina	Seawater	*A. butzleri*
		Seaweed	*A. butzleri*

ND: Not Detected.

**Table 3:** Antibiotic resistance of *A. butzleri* and *A. cryaerophilus* strains isolated from water samples and seaweeds (*n* = 28).

Antibiotics	Isolates from																								Total (*n* = 28)		
	Rivers (*n* = 9)			Streams (*n* = 2)			Ponds (*n* = 6)						Well Waters (*n* = 2)			Spring Waer (*n* = 1)			Seawater (*n* = 3)			Seaweed (*n* = 5)					
							*AB* (*n* = 5)			*AC* (*n* = 1)																	
	R	I	S	R	I	S	R	I	S	R	I	S	R	I	S	R	I	S	R	I	S	R	I	S	R	I	S
Amoxicillin-clavulanic acid (AMC)	7	0	2	2	0	0	5	0	0	1	0	0	2	0	0	1	0	0	3	0	0	5	0	0	26	0	2
Ampicillin (AMP)	9	0	0	2	0	0	5	0	0	1	0	0	2	0	0	1	0	0	3	0	0	5	0	0	28	0	0
Cefalotin (KF)	9	0	0	2	0	0	5	0	0	1	0	0	2	0	0	1	0	0	3	0	0	5	0	0	28	0	0
Cefotaxime (CTX)	9	0	0	2	0	0	5	0	0	1	0	0	2	0	0	1	0	0	3	0	0	5	0	0	28	0	0
Ciprofloxacin (CIP)	1	0	8	0	0	2	0	0	5	1	0	0	0	0	2	0	0	1	0	0	3	0	0	5	2	0	26
Erythromycin (E)	0	2	7	0	0	2	0	0	5	1	0	0	0	0	2	0	1	0	1	0	2	1	0	4	3	3	22
Gentamycin (CN)	0	0	9	0	0	2	0	0	5	0	0	1	0	0	2	0	0	1	0	0	3	0	0	5	0	0	28
Nalidixic acid (NA)	9	0	0	2	0	0	5	0	0	1	0	0	2	0	0	1	0	0	3	0	0	5	0	0	28	0	0
Streptomycin (S)	0	0	9	0	0	2	0	0	5	1	0	0	0	0	2	0	0	1	0	0	3	0	0	5	1	0	27
Tetracycline (TE)	9	0	0	2	0	0	5	0	0	1	0	0	2	0	0	1	0	0	3	0	0	5	0	0	28	0	0

R: resistant; I: intermediate; S: susceptible; *AB: A. butzleri*; *AC*: *A. cryaerophilus; n* indicates the number of isolates from each aquatic environment.

**Table 4:** Tetracycline resistance genes in nine strains isolated from water samples.

Sample	Specie	*tet* Resistance Genes
Seawater	*A. butzleri*	*tetW^−^, tetO^+^, tetA^−^*
River	*A. butzleri*	*tetW^+^, tetO^+^, tetA^−^*
Pond with aquatic animals	*A. butzleri*	*tetW^+^, tetO^−^, tetA^−^*
River	*A. butzleri*	*tetW^+^, tetO^+^, tetA^−^*
River	*A. butzleri*	*tetW^+^, tetO^+^, tetA^−^*
Seawater	*A. butzleri*	*tetW^+^, tetO^+^, tetA^−^*
River	*A. butzleri*	*tetW^+^, tetO^−^, tetA^−^*
Pond with turtles	*A. butzleri*	*tetW^+^, tetO^−^, tetA^−^*
Pond with aquatic animals	*A. cryaerophilus*	*tetW^+^, tetO^−^, tetA^−^*

**Table 5:** List of the primers used in this study.

Target Name	Primer Sequence (5′-3′)	Amplicon Size (bp)	Reference
*16s rDNA*	cggtgaatacgttcycgg	142	[63]
	gghtaccttgttacgactt		
*tetA*	gctacatcctgcttgccttc	210	[64]
	catagatcgccgtgaagagg		
*tetW*	acatcattgatactccaggtcacg	120	[51]
	tttcactttgtggttgaacccctc		
*tetO*	ggaggggttcaaccacaaag	88	[51]
	ctatgtaaataaaatggatag		
*gyrA*	tggattaaagccagttcatagaag	344	[29]
	tcatmgwatcatcataatttggwac		
*qnrS*	gacgtgctaacttgcgtgat	118	[59]
	tggcattgttggaaacttg		
*butz*	cctggacttgacatagtaagaatga	401	[64]
*arco*	cgtattcaccgtagcatagc		
*skir*	ggcgatttactggaacaca	641	[64]
*arco*	cgtattcaccgtagcatagc		
*cry1*	tgctggagcggatagaagta	257	[64]
*cry2*	aacaacctacgtccttcgac		

## Data Availability

Nucleotide sequence data reported in this study are available in the GenBank™ database under the accession numbers MW678780, MW683239, MW678840, MW678844, MW678845, MW683240 (*Arcobacter butzleri* 16S rRNA gene, partial sequence).
